# Physiological Effect of Cutting Height and High Temperature on Regrowth Vigor in Orchardgrass

**DOI:** 10.3389/fpls.2017.00805

**Published:** 2017-05-19

**Authors:** Gordon B. Jones, Jasper B. Alpuerto, Benjamin F. Tracy, Takeshi Fukao

**Affiliations:** ^1^Department of Crop and Soil Environmental Sciences, Virginia Tech, BlacksburgVA, United States; ^2^Translational Plant Sciences Program, Virginia Tech, BlacksburgVA, United States; ^3^Fralin Life Science Institute, Virginia Tech, BlacksburgVA, United States

**Keywords:** *Dactylis glomerata*, defoliation, heat stress, photosynthesis, energy reserves

## Abstract

Producers of orchardgrass (*Dactylis glomerata* L.) hay in the Mid-Atlantic US have experienced a reduction in regrowth vigor and a decline in the persistence of their swards. The common management practice for the region is to harvest the first growth of hay by cutting at 2.5–7.5 cm height in May or June. We hypothesize that high temperature and low cutting height interact to limit the regrowth rate. To test this, orchardgrass plants were cut to either 2.5 or 7.5 cm and then placed into environmentally controlled chambers with a constant temperature of 20 or 35°C. Stubble was harvested on days 0, 1, 3, and 11 following cutting and subjected to metabolite analysis. Photosynthetic parameters were measured in the regrown leaves on days 3 and 11, and regrowth biomass was recorded on day 11. Under optimal growth temperature (20°C), vegetative regrowth upon defoliation was significantly enhanced when more stubble tissue remained. However, this advantage was not observed under heat stress. Defoliation generally decreases the abundance of carbohydrate reserves in stubble. Interestingly, high temperature stimulated the accumulation of starch and ethanol-soluble carbohydrates in plants cut to 7.5 cm. The similar trends were also observed in protein, amino acids, nitrate, and ammonium. These responses were not pronounced in plants cut to 2.5 cm, presumably due to inhibited photosynthesis and photosystem II photochemistry. Overall, we anticipated that heat-activated metabolite accumulation is part of adaptive response to the stress. However, modified allocation of carbohydrate and nitrogen reserves leads to reduced vegetative regrowth upon defoliation. These data suggest that cutting height management for orchardgrass may be more effective for its regrowth vigor and productivity in cool seasons or when cool weather follows hay harvest.

## Introduction

Orchardgrass is a high yielding cool-season forage grass which is a valuable feedstuff for several classes of livestock. Globally, orchardgrass is the 4th most produced perennial cool-season forage grass seed and is widely planted in North America, Europe, and East Asia (Stewart and Ellison, 2011). Although orchardgrass was first cultivated and has been grown in the eastern United States since 1760 (Van Santen and Sleper, 1996), producers in this region have recently observed a decrease in the persistence and regrowth vigor of swards. Too low of a cutting height, a practice partially allowed by the adoption of disk-type hay mowers in the 1980s (Adams, 1996), has been implicated as a possible cause of the recent orchardgrass persistence problem.

Cutting height is a major determinant of quantity and quality of stubble from which the sward will regrow in perennial glasses. The energy for regrowth is supplied by carbohydrates generated in the remaining photosynthetic tissue and non-structural carbohydrates (NSC) stored in lower stems (Morvan-Bertrand et al., 1999; Donaghy et al., 2008; Alderman et al., 2011). Harvest at a low cutting height will remove the majority or all photosynthetic tissue and some of the stem tissue containing NSC, reducing the energy sources for regrowth. For regrowing tillers to survive and contribute to whole-plant photosynthesis, they must be supplied with adequate mineral nutrients and energy to grow leaf area and be recruited into the canopy (Ong, 1978; Davies, 1988). In perennial ryegrass, mRNA accumulation of a sucrose transporter gene, *LpSUT1*, is elevated in leaf sheaths upon defoliation (Berthier et al., 2009). Consistently, defoliation suddenly changes the activity of leaf sheath phloem from unloading to loading (Amiard et al., 2003). These results emphasize that the stubble tissue serves as a carbon source to support regrowth upon defoliation.

It is anticipated that high temperature influences the energy status of regrowing grasses following defoliation. Temperatures above optimum inhibit Photosystem II reactions and ATP synthesis on the thylakoid membrane and reduce Rubisco activase activity, resulting in lower photosynthetic activity in various plants (Havaux, 1996; Bukhov et al., 1999; Allakhverdiev et al., 2008). The optimal temperature of respiration is generally higher than that of photosynthesis (Yamori et al., 2014). Thus, carbohydrate consumption can be more active than carbohydrate synthesis under heat stress, leading to reduced accumulation of NSC in leaves (Brown and Blaser, 1970; Youngner and Nudge, 1976; Slack et al., 2000).

While previous studies have examined the effects of cutting height and temperature on orchardgrass and other cool-season grasses independently, we are not aware of any experiments to study these stress components together. Evaluation of stubble carbon and nitrogen status along with measurements of photosynthetic parameters in regenerated leaves provides a unique view into the physiological processes of regrowth in orchardgrass under supraoptimal temperature conditions and cut to a low cutting height. This information will help to explain management × environment interactions in orchardgrass hay production and will inform hay producers about the regrowth implications of defoliation height under differing temperature conditions.

## Materials and Methods

### Plant Growth and Stress Treatment

Seeds of orchardgrass (*Dactylis glomerata* L. cv. Benchmark Plus) were germinated in moist paper towels under ambient conditions for 3 days. Seedlings were transplanted to 2.25 L pots (13 cm diameter × 17 cm height) containing soil mix (Metro Mix 300; SunGro, Agawam, MA, United States) with three seedlings pot^-1^ (3 pots per treatment × 3 replicates). Plants were grown in the greenhouse at the Virginia Polytechnic Institute and State University (Blacksburg, VA, United States) at 28°C day/23°C night under ambient light conditions. Plants were watered daily to field capacity and supplied with 15 mL of slow-release fertilizer (Osmocote Plus; The Scotts Company, Marysville, OH, United States) twice during the establishment phase. After 10 weeks, plants were clipped to 10 cm and allowed to regrow. Plants were vernalized in the greenhouse during winter at 14–7°C under ambient light conditions for 8 weeks. Flowering was initiated with 23°C day/18°C night and supplemental lighting (14 h light/10 h dark), and plants were allowed to mature to anthesis—stage R4-R5 (Moore et al., 1991). Plants were then transferred to environmentally controlled chambers set to 20°C constant temperature for 5 days. Following this acclimatization, plants were cut to either 2.5 or 7.5 cm above the soil surface and were regrown at 20°C or 35°C constant temperature. Both chambers maintained 70% relative humidity, 14 h light/10 h dark at 400 μmol m^-2^ s^-1^ of photosynthetically active radiation. On days 0, 1, 3, and 11 days following cutting, stubble tissue was harvested, separated from regrowth, immediately frozen in liquid nitrogen, and stored at -80°C.

### Chlorophyll Fluorescence and Content Measurements

Chlorophyll florescence parameters were measured in regrowing leaf blades on days 3 and 11 using a chlorophyll fluorometer (MINI-PAM-II; WALZ, Effeltrich, Germany) and associated leaf-chip holder (2035-B). Leaf areas used for dark-adapted *F*_v_/*F*_m_ were exposed to dark conditions for 30 min using a dark leaf clip. Light adapted *F*_v_/*F*_m_ was determined after exposure to continuous actinic light (400 μmol m^-2^ s^-1^) for 5 min. Calculations for effective quantum yield of PS II photochemistry (*ϕ_PSII_*), photochemical quenching (*qP*), and non-photochemical quenching (*NPQ*) were (*F*′_m_-*F*′)/*F*′_m_, (*F*′_m_-*F*)/(*F*′_m_-*F*′_0_), and *F*_m_/*F*′_m_-1, respectively (Murchie and Lawson, 2013). Chlorophyll content was estimated in regenerating leaf blades using a portable chlorophyll meter (Opti-Sciences CCM-300, Hudson, NH, United States).

### Gas Exchange Measurements

On days 3 and 11 following defoliation, gas exchange measurements were made on regrowing leaf blades. A portable photosynthesis analysis system (LI-6400XT; LI-COR Lincoln, NE, United States) was used to determine the rates of CO_2_ assimilation, stomatal conductance, and transpiration. The system maintained the measurement conditions of 400 μmol CO_2_ mol^-1^ under 400 μmol m^-2^ s^-1^ of photosynthetically active radiation at 20°C and 60–70% relative humidity.

### Carbohydrate Assays

Water- and ethanol-soluble carbohydrates (WSC and ESC) and starch were analyzed using methods adapted from Fukao et al. (2012). Stubble tissue (30 mg) was homogenized in 1 mL of 80% (v/v) ethanol and incubated at 80°C for 20 min. After centrifugation, the supernatant was collected. The ethanol extraction was repeated twice more, and the three extracts were pooled. The pellet was then suspended in 1 mL water and incubated at 80°C for 20 min. Water extraction was repeated, and the supernatants from both water extractions were combined. The ethanol and water extracted solutions were dried using a vacuum concentrator and then re-dissolved in 1 mL of water. Soluble carbohydrates were measured by the anthrone method. Glucose was used as a standard. The carbohydrate extracts were incubated with 1 mL of 0.14% (w/v) anthrone solution in 100% sulfuric acid at 100°C for 20 min. Samples were cooled, and their absorbance at 620 nm was determined with a spectrophotometer. Starch content was quantified in the pellet used for soluble carbohydrate extraction. The pellet was re-suspended in 1 mL water containing 10 units of heat-resistant α-amylase. The suspension was incubated for 30 min at 95°C. The reactant was then mixed with 25 μL of 1M sodium citrate (pH 4.8) and 5 units of amyloglucosidase and incubated at 55°C for 1 h. The mixture was centrifuged for 30 min, and the glucose content of 100 μL of the supernatant was determined by the anthrone method as described above.

### Nitrate, Ammonium, and Total Amino Acid Assays

Frozen tissue (75 mg) was homogenized in 450 μL of 0.83 M perchloric acid on ice. After centrifugation, 300 μL of the supernatant was neutralized with 75 μL of 1 M bicin (pH 8.3) and 70 μL of 4 M potassium hydroxide. Following centrifugation, the supernatant was used for nitrate, ammonium, and amino acid assays as described in van Veen et al. (2013) and Alpuerto et al. (2016). For nitrate, the extract (10 μL) was incubated with 40 μL of 5% (w/v) salicylic acid in 100% sulfuric acid at 25°C for 20 min. The solution was mixed with 950 μL of 2 M sodium hydroxide, and the absorbance at 410 nm was determined with a spectrophotometer. Potassium nitrate was used as the standard. For ammonium, 25 μL of the extract was mixed with 375 μL of 8.8% (w/v) salicylic acid, 10 M sodium hydroxide, 21.5 mM ethylenediaminetetraacetic acid (EDTA), and 6.7 mM sodium nitroferricyanide (III) dehydrate. The mixture was added to 625 μL of 70 mM sodium phosphate, monobasic (NaH_2_PO_4_) and 45 mM sodium dichlorisocyanurate and incubated for 2 h at 25°C. Following incubation, the absorbance at 660 nm was determined. Ammonium sulfate was used as the standard. For total amino acid, 80 μL of the extract was added to 20 μL of 3 M magnesium oxide and incubated in an opened 1.5 mL tube for 16 h at 25°C. After incubation, 80 μL of the solution was mixed with 50 μL of 0.2 mM sodium cyanide resolved in 8 M sodium acetate and 50 μL of 168 mM ninhydrin resolved in 100% 2-methoxyethanol. The mixture was incubated at 100°C for 15 min, and 1 mL of 50% isopropanol was immediately added to the solution. After cooling, the absorbance at 570 nm was measured with a spectrophotometer. Glycine was used as the standard.

### Protein Assay

Total protein was extracted from 50 mg of stubble tissue in a buffer containing 50 mM Tris-HCl (pH 8.0), 150 mM sodium chloride, 2 mM EDTA, 10% (v/v) glycerol, 0.5% (v/v) IGEPAL CA-360, and 1 mM phenylmethanesulfonyl fluoride on ice. Protein concentration was determined by Coomassie Plus protein assay reagent (Thermo Fisher Scientific, Waltham, MA, United States). Bovine serum albumin was used as the standard.

### Statistical Analysis

Statistical analysis was conducted using JMP Pro (ver. 11.0.0; SAS Institute, Cary, NC, United States). ANOVA was used to determine treatment differences at each time point. Tukey’s honest significant difference (HSD) was used to determine mean separation (α < 0.05).

## Results

### Low Cutting Height and High Temperature Suppress Regrowth upon Defoliation

**FIGURE 1:**
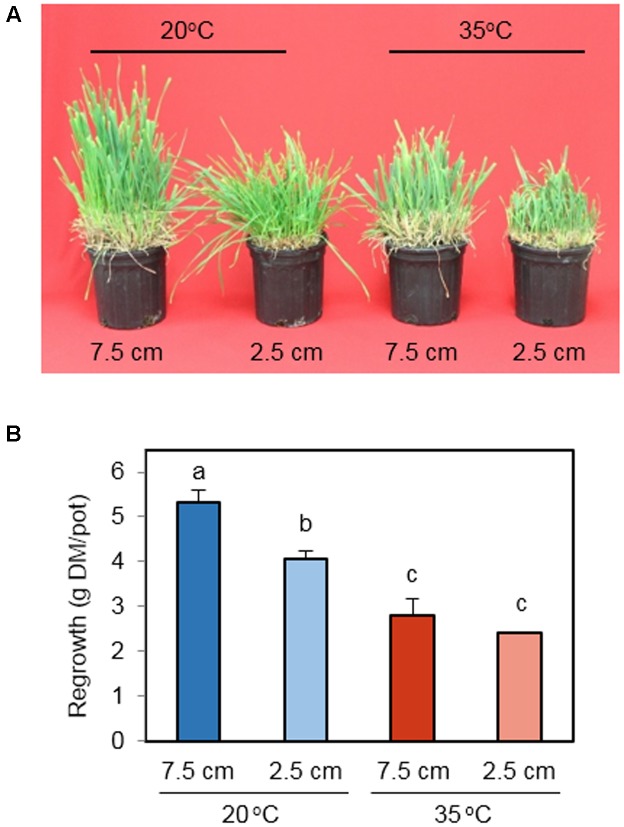
**The effect of cutting height and high temperature on regrowth of leaves from the stubble. (A)** Photos of orchardgrass 11 days after defoliation. Orchardgrass was exposed to clipping at 7.5 or 2.5 cm cutting height at the heading stage and placed under 20 or 35°C for 11 days. **(B)** Dry biomass of regrown leaves 11 days following defoliation. Data represent mean ± SE (*n* = 3). Bars not sharing the same letter are significantly different (*P* < 0.05).

This study broadly simulated the environmental conditions during regrowth following the first harvest of spring growth for orchardgrass hay/silage. Plants were excised at the early heading stage at 7.5 or 2.5 cm above the soil surface – a commonly recommended growth stage and cutting height range for hay harvest. Following defoliation, plants were regrown under control (20°C) or high temperature (35°C) conditions for up to 11 days. On day 11 the most vigorous regrowth was observed for the 20°C treatment cut to 7.5 cm with diminishing regrowth for the 20°C – 2.5 cm, 35°C – 7.5 cm, and 35°C – 2.5 cm treatments (**Figure 1A**). Consistently, the biomass of regrown tissue differed significantly with cutting height and temperature treatments (**Figure 1B**). At optimal temperature (20°C), 7.5 cm defoliation height supported more regrowth than 2.5 cm. Under heat stress (35°C), however, the advantage of high cutting height on regrowth vigor was not observed. When plants were cut to 7.5 cm, high temperature significantly suppressed regrowth. This temperature effect was also detected at 2.5 cm cutting height. These results indicate that heat stress affects biomass yield at both cutting heights, whereas the impact of cutting height is apparent only under non-stress temperature.

### The Influence of Defoliation Height and Heat Stress on Photosynthetic Parameters in Regenerating Leaves

**FIGURE 2:**
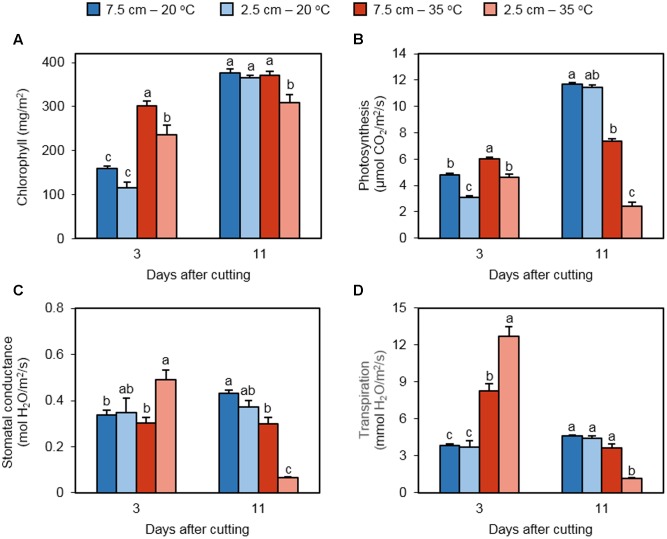
**The effect of cutting height and high temperature on photosynthetic parameters in re-emerging leaves following defoliation.** Orchardgrass was exposed to clipping at 7.5 or 2.5 cm cutting height at the heading stage and placed under 20 or 35°C for regrowth. After 3 or 11 days, chlorophyll **(A)**, photosynthesis **(B)**, stomatal conductance **(C)**, and transpiration **(D)** were monitored in newly emerged leaves. Data represent mean ± SE [*n* = 20 in **(A)**; *n* = 18 in **(B–D)**; 6–7 measurements per pot]. Bars not sharing the same letter are significantly different (*P* < 0.05).

Chlorophyll abundance in regrowing laminar tissue was quantified as a determinant of photosynthetic performance (**Figure 2A**). Three days following defoliation, chlorophyll was significantly greater in 35°C treatments as compared to 20°C treatments, irrespective of cutting height. In addition, chlorophyll abundance was reduced by low cutting height in heat-treated plants, but not in non-stressed plants. On day 11 following defoliation, a lower chlorophyll abundance was measured in the 35°C – 2.5 cm, but the other treatments did not differ.

Direct measurements of leaf gas exchange determined photosynthetic activity as well as stomatal conductance and transpiration rate in regrowing leaves. On day 3, the photosynthetic rate was greater at 35°C than 20°C in plants cut to 7.5 cm (**Figure 2B**). The same pattern was observed in plants cut to 2.5 cm. We also observed that short cutting height reduced photosynthetic activity in regrowing leaves under both non-stress and high temperature conditions. On the 11th day of recovery, photosynthesis rates were clearly augmented in plants cut to 7.5 and 2.5 cm at 20°C, and their values did not differ significantly. High temperature suppressed photosynthetic activity at both mowing heights, with a more severe reduction in plants trimmed to 2.5 cm. Stomatal conductance of regrowing leaves was significantly increased by low cutting height only at 35°C, not at 20°C on day 3 (**Figure 2C**). High temperature did not affect stomatal conductance at either cutting height. On day 11, heat stress repressed stomatal conductance at both mowing heights. However, the negative effect of low cutting height on stomatal conductance was detected only under high temperature. On day 3, transpiration was stimulated in response to high temperature at both defoliation levels, with higher induction in plants cut to 2.5 cm (**Figure 2D**). At 20°C, cutting height did not influence transpiration. On day 11, heat-induced transpiration was not observed at either mowing level; the 35°C – 2.5 cm treatment had a significantly lower transpiration rate than the other treatments.

**FIGURE 3:**
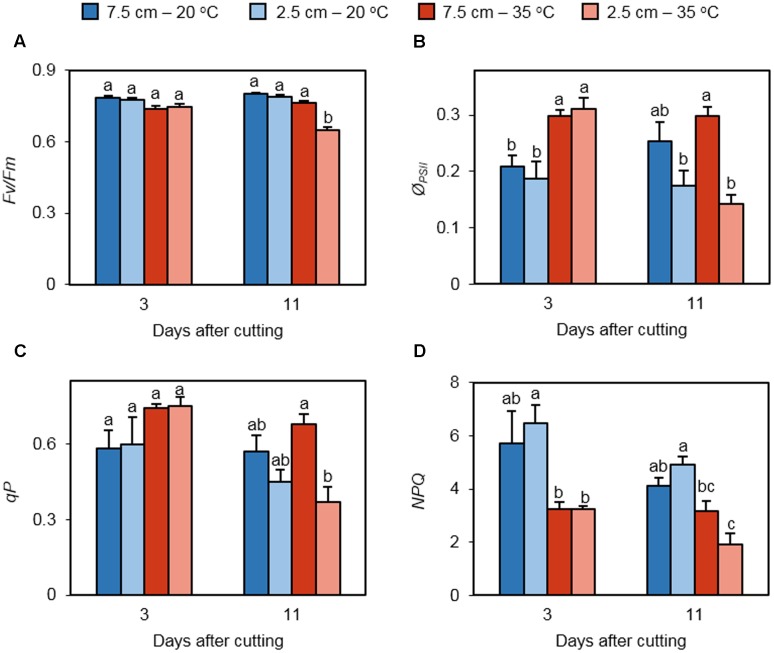
**The effect of cutting height and high temperature on photosystem II photochemistry in re-emerging leaves following defoliation.** Orchardgrass was exposed to clipping at 7.5 or 2.5 cm cutting height at the heading stage and placed under 20 or 35°C for regrowth. After 3 or 11 days, dark-adapted *F*_v_*/F*_m_ **(A)**, effective quantum yield of photosystem II (*Ø_PSII_*; **B**), photochemical quenching (*qP*; **C**), and non-photochemical quenching (*NPQ*; **D**) were monitored in newly emerged leaves. Data represent mean ± SE (*n* = 4; 1–2 measurements per pot). Bars not sharing the same letter are significantly different (*P* < 0.05).

Chlorophyll fluorescence measurements provided the insight into the performance of PSII in regrowing leaves under non-stress and heat stress conditions. Maximum quantum efficiency of dark-adapted PSII photochemistry (*F*_v_/*F*_m_) did not differ among treatments on day 3, but it was significantly lower in leaves of the 35°C – 2.5 cm treatment on day 11 (**Figure 3A**). The operating efficiency of PSII (*ϕ_PSII_*) was enhanced by high temperature in plants cut to 7.5 and 2.5 cm on day 3 but did not differ between the cutting heights (**Figure 3B**). On day 11, the positive effect of high temperature on *ϕ_PSII_* was canceled at both defoliation levels. Low cutting height suppressed *ϕ_PSII_* under heat stress. *qP* did not differ among the treatments on day 3 (**Figure 3C**). On day 11, *qP* was reduced at low mowing height only at high temperature. Heat stress restrained *NPQ* only in plants cut to 2.5 cm on day 3 (**Figure 3D**). This trend was also observed on day 11.

### The Impact of Cutting Height and Heat Stress on Carbohydrate Accumulation in Stubble

**FIGURE 4:**
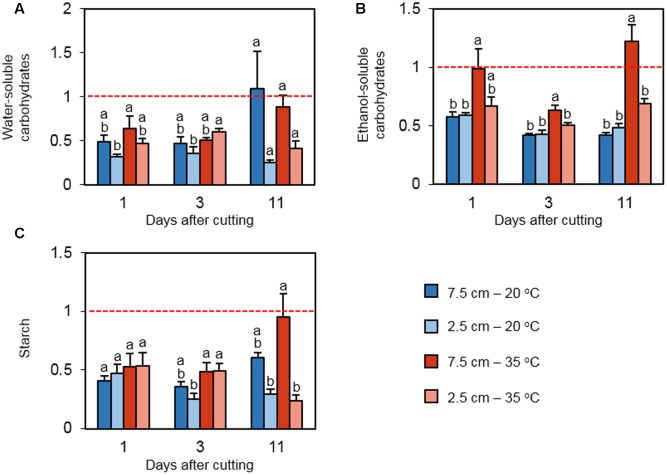
**The effect of cutting height and high temperature on the alterations in carbohydrate reserves in stubble during recovery from defoliation.** Orchardgrass was exposed to clipping at 7.5 or 2.5 cm cutting height at the heading stage and placed under 20 or 35°C for regrowth. After 0, 1, 3, or 11 days, the concentrations of major carbon reserves were quantified in stubble. Leaf tissue newly emerged during regrowth was not included in this analysis. Data represent the change in water-soluble carbohydrates **(A)**, ethanol-soluble carbohydrates **(B)**, and starch **(C)** relative to their levels on day 0 (Dashed red line). The error bars indicate SE (*n* = 9). Bars not sharing the same letter are significantly different (*P* < 0.05).

Concentrations of three classes of carbohydrates were measured to elucidate how cutting height and heat stress influence the status of carbon reserves in the stubble during regrowth following defoliation. WSC extracts contain primarily fructan, while ESC extracts include mono- and disaccharides (Kagan et al., 2014). On days 1 and 3 following defoliation, the levels of WSC were low relative to the values on day 0 in all treatments (**Figure 4A**). The effect of cutting height and temperature on relative WSC concentration was not detected on day 1. However, on day 3, relative WSC content was greater under high temperature in plants cut to 2.5 cm. On day 11, no significant difference among the relative WSC concentrations was detected. The relative concentration of ESC was positively affected by high temperature only in plants cut to 7.5 cm on day 1 (**Figure 4B**). The effect of high temperature on ESC continued on days 3 and 11. In addition, low cutting height suppressed the relative ESC content under high temperature at these time points. For relative starch concentration, there was no significant difference among the treatments on day 1 (**Figure 4C**). On day 3, the relative starch concentration was greater at high temperature only when cut to 2.5 cm, which is consistent with the observation in WSC. This trend was not observed on day 11, but low defoliation height suppressed the relative starch content at high temperature, which is in accordance with the observation in ESC at the same time point.

### Cutting Height and High Temperature Affect the Abundance of Nitrogen Compounds in Stubble

**FIGURE 5:**
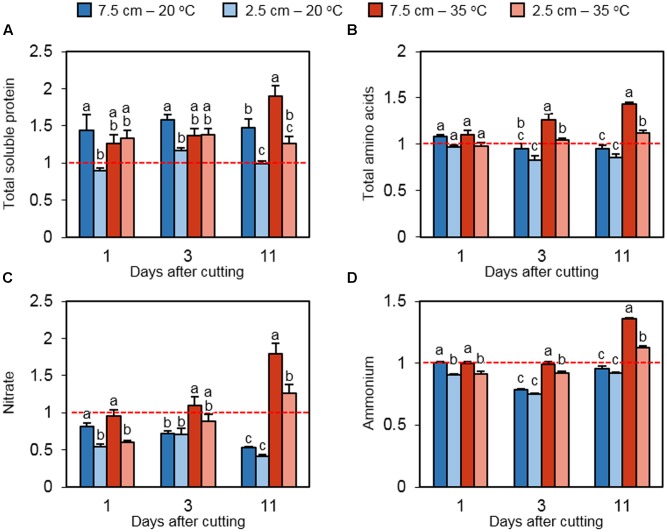
**The effect of cutting height and high temperature on the alterations in nitrogen reserves in stubble during recovery from defoliation.** Orchardgrass was exposed to clipping at 7.5 or 2.5 cm cutting height at the heading stage and placed under 20 or 35°C for regrowth. After 0, 1, 3 or 11 days, the concentrations of major N reserves were quantified in stubble. Leaf tissue newly emerged during regrowth was not included in this analysis. Data represent the changes in total protein **(A)**, total free amino acids **(B)**, nitrate **(C)**, and ammonium **(D)** relative to their levels on day 0 (Dashed red line). The error bars indicate SE [*n* = 9 in **(A,C,D)**; *n* = 6 in **(B)**]. Bars not sharing the same letter are significantly different (*P* < 0.05).

To determine the status of nitrogen reserves in stubble tissue, the relative concentrations of total soluble protein, total free amino acids, nitrate, and ammonia were measured. At all of the three time points, high cutting height increased the relative protein content at 20°C (**Figure 5A**). Under high temperature, the positive effect of high cutting height was observed only on day 11. The protein content was also induced by high temperature only in plants cut to 7.5 cm on day 11. Relative total amino acids did not differ significantly among treatments on day 1 (**Figure 5B**). On days 3 and 11, high temperature increased the abundance of total amino acids at both mowing levels. However, low cutting height limited the accumulation of relative total amino acids under heat stress at these time points. Relative nitrate concentrations were suppressed by low cutting height under non-stress and heat stress conditions on day 1 (**Figure 5C**). This effect is not observed on day 3, but high temperature increased the relative nitrate content in plants cut to 7.5 cm. The positive effect of high temperature on nitrate was also detected on day 11 at both defoliation levels. Ammonium concentrations followed a similar pattern to that of nitrate (**Figure 5D**).

## Discussion

Supraoptimal temperatures reduce the growth rate and biomass accumulation of various C_3_ species (Youngner and Nudge, 1976; Rutledge et al., 2012; Song et al., 2014). We also observed that heat stress significantly suppressed the biomass yield following defoliation both in plants cut to 7.5 and 2.5 cm (**Figure 1**). Cutting height of forage grasses is a critical factor determining hay/silage yield and subsequent productivity (Mislevy et al., 1977; Sheffer et al., 1978; Fulkerson and Michell, 1987; Garay et al., 1999). In this study, increased mowing height improved regrowth of orchardgrass at 20°C. However, the positive effect of high cutting height was canceled under heat stress. These results were in accordance with the observation by Youngner and Nudge (1976) who grew Kentucky bluegrass at 21 and 32°C with 2 and 3.75 cm cutting heights. They found that taller stubble tissue left following defoliation resulted in greater biomass regrowth at the lower air temperature but not at the higher. These data suggest that cutting height management for C_3_ grasses may be more effective in increased regrowth vigor and productivity during cool seasons or when cool weather follows hay harvest.

Chlorophyll content is an important component that affects photosynthetic capacity in plants. Studies of heat stress in cool-season grasses reported a reduction in chlorophyll content caused by high temperature in wheat (Pradhan et al., 2012) and bentgrass (*Agrostis spp.*) (Liu and Huang, 2000; Jespersen et al., 2016). However, Yu et al. (2014) found that undefoliated, heat-stressed (35°C) tall fescue had higher chlorophyll content than control plants (25°C) during the initial 21 days of heat stress. Consistently, we observed that plants regrown at 35°C contained more chlorophyll than those at 20°C (**Figure 2A**). This initial rise in leaf chlorophyll could be caused by an increased rate of substrate mobilization from the stubble tissue as a result of the elevated temperature. It appears that cutting height is also crucial for chlorophyll generation in regrowing leaves. Indeed, low defoliation height suppressed the amount of chlorophyll under high temperature on days 3 and 11 (**Figure 2A**). A study manipulating cutting height in frequently mown bermudagrass (*Cynodon dactylon* L.) found increased total shoot chlorophyll in the higher cutting height treatments when measured after 4 weeks (Bunnell et al., 2005).

A reduction in net photosynthesis has been observed at supraoptimal temperatures in numerous C_3_ turf and forage grass species including Kentucky bluegrass (*Poa pratensis* L) (Song et al., 2014), tall fescue [*Schedonorus phoenix* (Scop.) Holub] (Jiang and Huang, 2001; Yu et al., 2014), Chinese rye grass (*Leymus chinensis* L.) (Xu and Zhou, 2006), perennial ryegrass (*Lolium perenne* L.) (Jiang and Huang, 2001), and creeping bentgrass (*Agrostis stolonifera* L.) (Pote et al., 2006). We found that photosynthetic rate initially increased on day 3 for the 35°C treatments as compared to the 20°C counterparts, which is likely related to leaf chlorophyll content (**Figures 2A,B**). On day 11, heat stress did not alter the abundance of chlorophyll in plants cut to 7.5 cm. However, net photosynthesis was significantly suppressed by high temperature in the same plants. Removal of more vegetative tissue (2.5 cm cutting height) further reduced photosynthesis under heat stress. This can be caused by the observed reduction in stomatal conductance (**Figure 2C**) and presumably increased photorespiration under prolonged heat stress. Increased transpiration is an adaptation response to heat stress wherein transpirational cooling reduces the surface temperature in leaves (Jagadish et al., 2015). We observed that the rate of transpiration was elevated in response to heat on day 3 regardless of defoliation height (**Figure 2D**). Greater transpiration in plants at low cutting height may reflect more severe heat stress in leaves that emerged from the shorter stubble. Under prolonged heat stress (day 11), increased transpiration was not detected; low mowing height even reduced the rate of transpiration. It seems that the cooling effect from elevated transpiration does not last for a prolonged period of heat.

The functioning of photosystem II is an indicator of plant stress and a determinant of photosynthetic performance. High temperature stress has been shown to reduce the maximum efficiency of PSII photochemistry (*F*_v_/*F*_m_) and effective quantum yield of photosystem II *(Φ_PSII_*) in C_3_ plants (Liu and Huang, 2000; Jiang and Huang, 2001; Xu and Zhou, 2006; Wang et al., 2010). In this study, we found that *F*_v_/*F*_m_ was reduced for the 35°C – 2.5 cm treatment on day 11 (**Figure 3A**). Heat stress for 3 days increased *Φ_PSII_* regardless of cutting height (**Figure 3B**). This changing pattern was similar to that in leaf chlorophyll content at day 3 (**Figure 2A**), suggesting that the amount of chlorophyll can be a major cause for heat-activated quantum yield of photosystem II at this time point. Under prolonged high temperature, low cutting height reduced *Φ_PSII_*_,_ which is consistent with the observations in chlorophyll, net photosynthesis, stomatal conductance, transpiration, *F*_v_/*F*_m_, and *qP* (**Figures 2, 3A,C**). These data indicate that defoliation height or the amount of stubble tissue remained following defoliation significantly influences photosynthetic capability, stomatal regulation, and photosystem II photochemistry in newly emerged leaves under prolonged heat stress. In general, *NPQ* is elevated with increasing temperature in leaves of non-defoliated plants (Haldimann and Feller, 2004; Salvucci and Crafts-Brandner, 2004). However, we observed that NPQ was reduced at 35°C in leaves regrowing from the plants cut to 2.5 cm on days 3 and 11 (**Figure 3D**). Leaves emerging from defoliated and non-defoliated plants may respond differentially to high temperature in terms of NPQ-dependent stress adaptation.

Heat stress generally reduces NSC contents in leaves of non-defoliated C_3_ plants (Brown and Blaser, 1970; Youngner and Nudge, 1976; Slack et al., 2000). However, we observed that high temperature induced the accumulation of ESC in the stubble of plants cut to 7.5 cm. Under the stress, starch content was also greater in the 7.5 cm treatment than the 2.5 cm treatment. The abundance of soluble carbohydrates and starch is positively correlated with the degree of heat tolerance in tomato (Pressman et al., 2002; Firon et al., 2006). In rice, continuous mild heat stimulates sugar transporters in reproductive organs, resulting in increased starch accumulation in pollen (Chung et al., 2014). Based on these data, induction of carbohydrate accumulation under prolonged heat stress in stubble tissue may be an adaptive response to the stress in defoliated plants. However, the modification of carbohydrate allocation can lead to reduced regeneration of vegetative tissue under high temperature. Heat-mediated accumulation of carbohydrates was not obvious when most photosynthetic tissue was removed (2.5 cm cutting height), probably due to the lack of or reduced carbon assimilation capability.

As observed in carbohydrate assays, prolonged heat stress induced the accumulation of protein, amino acids, nitrate, and ammonium in the stubble of plants cut to 7.5 cm (**Figure 5**). Shorter cutting height limited these stress responses, but we still observed significant elevations in the amino acid, nitrate, and ammonium contents as compared to its counterpart (20°C – 2.5 cm treatment). Free amino acids are known to accumulate during heat and other abiotic stresses such as drought and flooding for membrane stabilization, free radical scavenging, and osmotic adjustment (Rai, 2002; Seki et al., 2007; Du et al., 2011; Rutledge et al., 2012; Tamang et al., 2014; Alpuerto et al., 2016). An increase in amino acid content in stubble would be an acclimation response to the stress. However, the significance of heat-induced nitrate and ammonium accumulation in stress adaptation is unknown. It is expected that promoted preservation of major nitrogen compounds in stubble can lead to reduced translocation of these reserves into sink tissue, resulting in slow vegetative regrowth.

## Conclusion

At optimal temperature for cool-season grasses (20°C), leaving more stubble tissue resulted in greater biomass regrowth upon defoliation. However, this advantage was negated under prolonged heat stress (35°C). It appears that high temperature triggers changes in carbon and nitrogen allocation in stubble, resulting in the accumulation of mono- and disaccharides, starch, amino acids, nitrate, and ammonium in the source tissue. This can contribute to the enhanced protection in stubble against heat stress, but inhibit the translocation of energy resources into growing leaves (sinks). Heat-induced accumulation of carbohydrates and nitrogen compounds were more prominent in plants cut to 7.5 cm than 2.5 cm, which can explain in part the cancelation of the positive effect of high cutting height on vegetative regrowth. It is likely that greater net photosynthesis and photosystem II photochemistry in taller stubble under high temperature can facilitate the metabolic adjustment to the stress but do not benefit the formation of new leaves. Comparative analysis of orchardgrass accessions with contrasting heat tolerance can determine how tolerant genotypes manage carbohydrate and nitrogen allocation and metabolism under the stress.

## Author Contributions

GJ and TF designed research. GJ, JA, and TF performed research and analyzed data. GJ wrote the draft paper, which was revised by BT and TF.

## Conflict of Interest Statement

The authors declare that the research was conducted in the absence of any commercial or financial relationships that could be construed as a potential conflict of interest.
